# The effect of 12 weeks of mechanical vibration on root resorption: a micro-CT study

**DOI:** 10.1186/s40510-021-00369-1

**Published:** 2021-08-23

**Authors:** Hakan Yilmaz, Fethiye Cakmak Ozlu, Tamer Turk, Mehmet Ali Darendeliler

**Affiliations:** 1 Department of Orthodontics, Faculty of Dentistry, Yeditepe University, Istanbul, Turkey; 2 Department of Orthodontics, Faculty of Dentistry, Ondokuz Mayıs University, Samsun, Turkey; 3 Department of Orthodontics, Faculty of Dentistry, The University of Sydney, Sydney, Australia

## Abstract

**Objective:**

The aim was to investigate the effect of mechanical vibration on root resorption with or without orthodontic force application.

**Material and methods:**

Twenty patients who required maxillary premolar extractions as part of orthodontic treatment were randomly divided into two groups of 10: no-force group and force group. Using a split-mouth procedure, each patient’s maxillary first premolar teeth were randomly assigned as either vibration or control side for both groups. A buccally directed vibration of 50 Hz, with an Oral-B HummingBird device, was applied to the maxillary first premolar for 10 min/day for 12 weeks. After the force application period, the maxillary first premolars were extracted and scanned with micro-computed tomography. Fiji (ImageJ), performing slice-by-slice quantitative volumetric measurements, was used for resorption crater calculation. Total crater volumes were compared with the Wilcoxon and Mann–Whitney U tests.

**Results:**

The total crater volumes in the force and no-force groups were 0.476 mm^3^ and 0.017 mm^3^ on the vibration side and 0.462 mm^3^ and 0.031 mm^3^ on the control side, respectively. There was no statistical difference between the vibration and control sides (*P* > 0.05). There was more resorption by volume in the force group when compared to the no-force group (*P* < 0.05).

**Conclusion:**

Mechanical vibration did not have a beneficial effect on reducing root resorption; however, force application caused significant root resorption.

## Introduction

Orthodontically induced inflammatory root resorption (OIIRR) is an important negative sequela and undesirable risk of orthodontic treatment [1, 2]. Although research has attempted to define etiological factors, OIIRR can occur due to a number of factors, including patient-related genetic factors, non-patient-related orthodontic forces, and iatrogenic factors [3]. Studies reported that OIIRR prevalence is between 73 and 100% [3, 4]. Fortunately, in most cases, OIIRR is insignificant and does not have any effect on the survival or functionality of the affected teeth on a long-term basis [5]. Nevertheless, in some cases, a reduced crown to root ratio due to severe root resorption may have a significant impact on the prognosis, specifically in the presence of periodontal problems or trauma [6]. The literature states that 1 to 5% of teeth exposed to orthodontic force have severe OIIRR (exceeding 4 mm or one-third of the original root length) [7].

The practice of mechanical vibration with orthodontic treatment has been recommended as a way to reduce the duration of treatment and root resorption, but there is inadequate evidence to support this claim [8]. In medicine, mechanical vibration has been shown to be beneficial in slowing down the rate of bone atrophy in osteoporosis, accelerating the healing process of fractures, increasing bone remodeling, and restoring muscle strength, balance, and mobility [9–11]. In dentistry, Darendeliler et al. [12] and Nishimura et al. [13] used mechanical vibration in orthodontic practice and stated that the rate of tooth movement was accelerated with mechanical vibration. In addition, it was suggested that mechanical vibration reduces pain caused by orthodontic force [12, 14]. Nevertheless, other studies found no evidence that mechanical vibration can significantly increase the rate of tooth movement [15–17] or reduce pain resulting from the orthodontic force [18, 19].

Mechanical vibration distributes the stress concentration in the periodontal ligament (PDL) with enhanced receptor activator of nuclear factor kappa-Β ligand (RANKL) expression of fibroblasts and osteoclasts [13, 20]; this process may also suggest the possibility of reducing OIIRR. Nevertheless, only two studies examined mechanical vibration in terms of OIIRR. DiBiase et al. [21] indicated that mechanical vibration did not affect OIIRR associated with the maxillary central incisor, but they used periapical radiographs to determine the amount of resorption. However, the quality and accuracy of periapical radiographs can be affected by several factors, primarily the orientation of the film and the placement of the X-ray tube [22]. Similar to DiBiase et al., Nishimura et al. [13] investigated mechanical vibration in rat molars with histological examination and noted that mechanical vibration did not affect OIIRR. As mentioned above, the number of studies is not adequate to prove whether mechanical vibration in terms of OIIRR is beneficial or not.

The aim was to examine the effect of mechanical vibration on root resorption of maxillary first premolars with a buccally directed force of 150 g or no force. The null hypothesis was that mechanical vibration would not affect the extent of root resorption. The alternative hypothesis was that the buccally directed controlled orthodontic force would increase the extent of root resorption on maxillary first premolars.

## Material and method

Forty maxillary first premolars from 20 orthodontic patients (7 boys and 13 girls; range 15.08–18.58 years; mean: 16.77 years) who had an indication for bilateral maxillary first premolar extraction as part of their orthodontic treatment composed this study. These patients were enrolled according to strict selection criteria as described previously [20, 23, 24]: permanent dentition, completion of apexification and no root blunting; similar crowding on each side of the maxillary arch; no previous orthodontic or orthopedic treatment; no unilateral or bilateral posterior cross-bites; no craniofacial anomaly; no history of trauma, bruxism, and parafunction; no periodontal disease; and no significant medical history that would affect the development or structure of the teeth and jaws and any subsequent tooth movement. The sample size was calculated by using Piface 1.72 and guaranteed 82.56% power. This number was reached by considering the standard deviation of 0.46 mm^3^ in a previous study [23]. The true difference of the means was estimated at 0.2 mm^3^, and type I error (α) was accepted as the standard value 0.05.

Ethics approval was attained from the Ethics Committee of Bulent Ecevit University (2012/23). After receiving verbal and written explanations, the subjects and their guardians consented to take part in this study. The trial was registered with the ClinicalTrials.gov supported by the US National Library of Medicine (NCT04686617).

On the study model of the maxillary jaw, the transpalatal arch was formed by bending a 0.09-mm steel wire between the right and left first molar teeth. Occlusion rising acrylic plates were added to the transpalatal arch to prevent potential contact of the teeth during buccal movement. These acrylic plates were of a mean 2 mm thickness. After fitting the transpalatal arches, occlusion rising acrylic plates were bonded with light-cured glass ionomer cement (Transbond™ Plus, 3M Unitek, Monrovia, USA) on the maxillary first molar teeth of all patients. After these procedures, the patients were randomly separated into two equal groups. Randomization was made using the Excel program (Microsoft, Redmond, WA, USA), and allocation was hidden in consecutively numbered, closed envelopes. Blinding was used for outcome assessments.

**Fig. 1:**
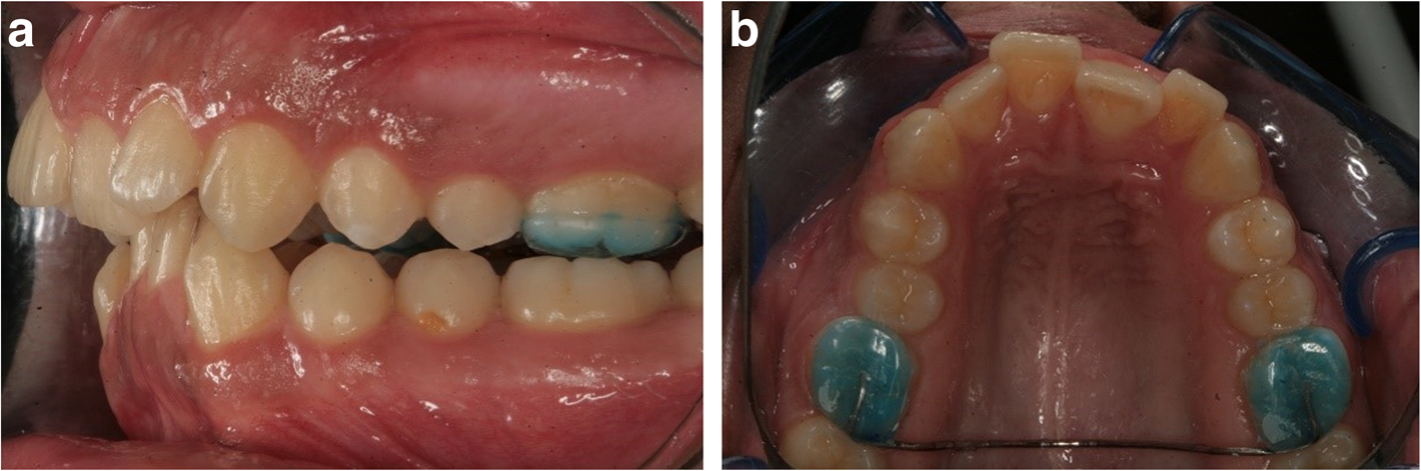
Transpalatal arch (no-force group)

**Fig. 2:**
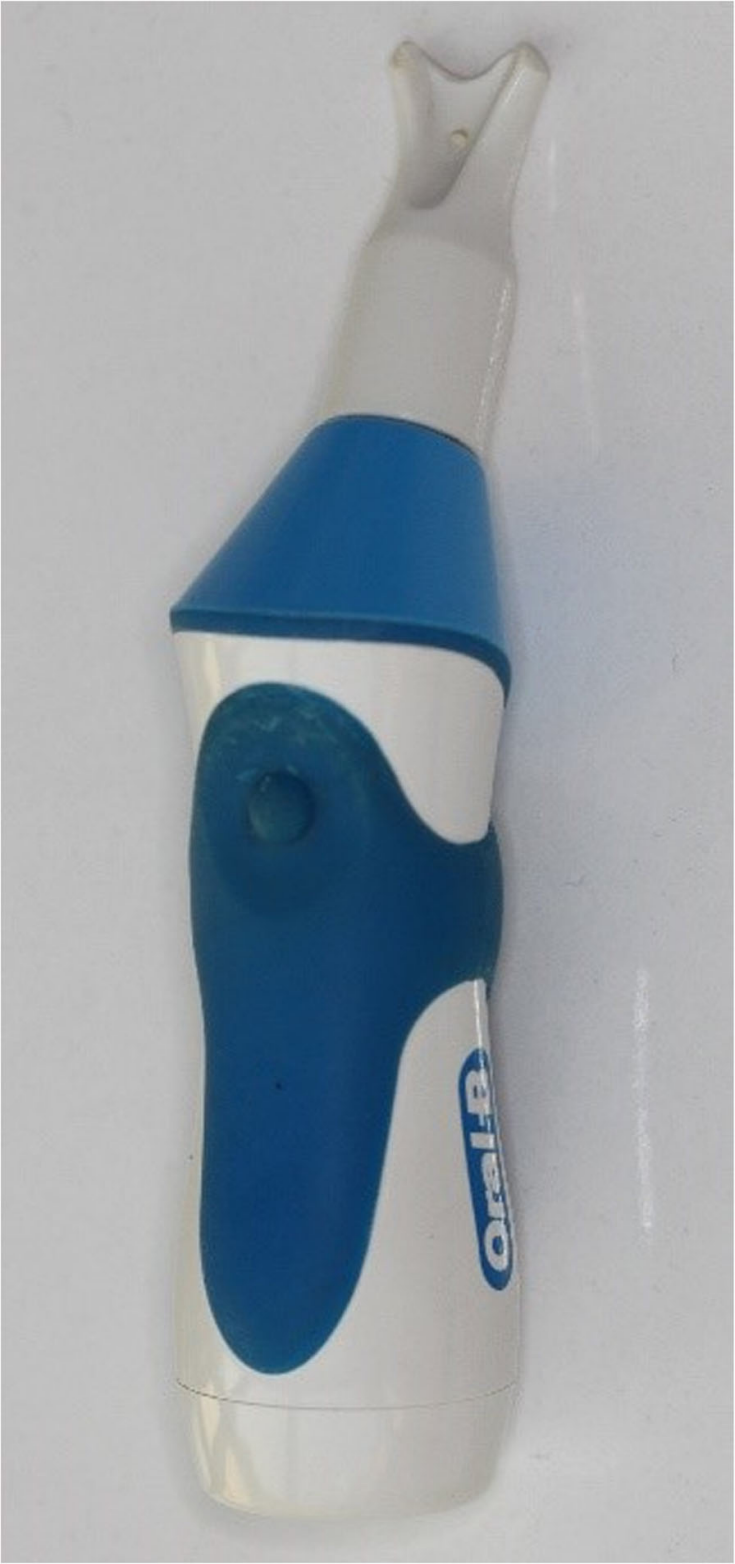
Oral-B HummingBird device with a modified tip

No-force group: the first premolar teeth on the right and left sides were randomly assigned (split-mouth design). Mechanical vibration was applied in the buccal direction on one side, and the other side served as the control (Fig. 1). Oral-B HummingBird device (Procter & Gamble, USA) with a modified tip was used for the application of vibration (Fig. 2). The tip was positioned mid-buccally of the teeth to perform a buccally directed vibration. Liao et al. [20] used the same device and stated that the vibrating frequency of the terminal tip was measured as 50 Hz. The vibration procedure was applied for 10 min/day for a period of 12 weeks. At the end of the 12th week, the first premolar teeth were extracted.

**Fig. 3:**
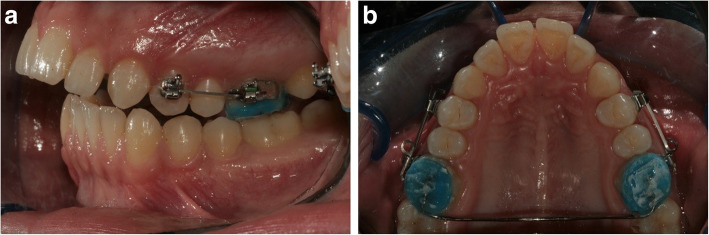
Transpalatal arch and TMA springs (force group)

Force group: The right and left side first premolar teeth were randomly assigned (split-mouth design). Mechanical vibration was applied in the buccal direction to one side, and the other side was used as the control. As in the no-force group, the same procedure was used for the application of vibration. Self-ligating speed (Strite Industries, Cambridge, Ontario, Canada) tubes and brackets with 0.022 × 0.026 in. slots were bonded to the buccal surfaces of the right and left first molar and first premolar teeth. A buccally directed force of 150 g, produced by a 0.017 × 0.025 in. beta-titanium-molybdenum alloy (TMA) (3M Unitek, Monrovia, CA) cantilever spring, was applied to the premolar teeth on both sides (Fig. 3). The force magnitude was measured with a strain gauge (Dentaurum). At the end of the 12th week, the first premolar teeth were extracted.

The premolar teeth were extracted under local anesthesia by the same dental practitioner in all cases. To remove blood and tissue remnants from the teeth after extraction, they were washed with a non-pressurized isotonic solution without touching the root surfaces, then each tooth was placed in a 5-ml sterile tube containing 10% formalin solution (Sarstedt Ag & Co., Nümbrecht, Germany). After 2 weeks, the formalin solution was changed and no other procedure was applied until examination of the roots.

**Fig. 4:**
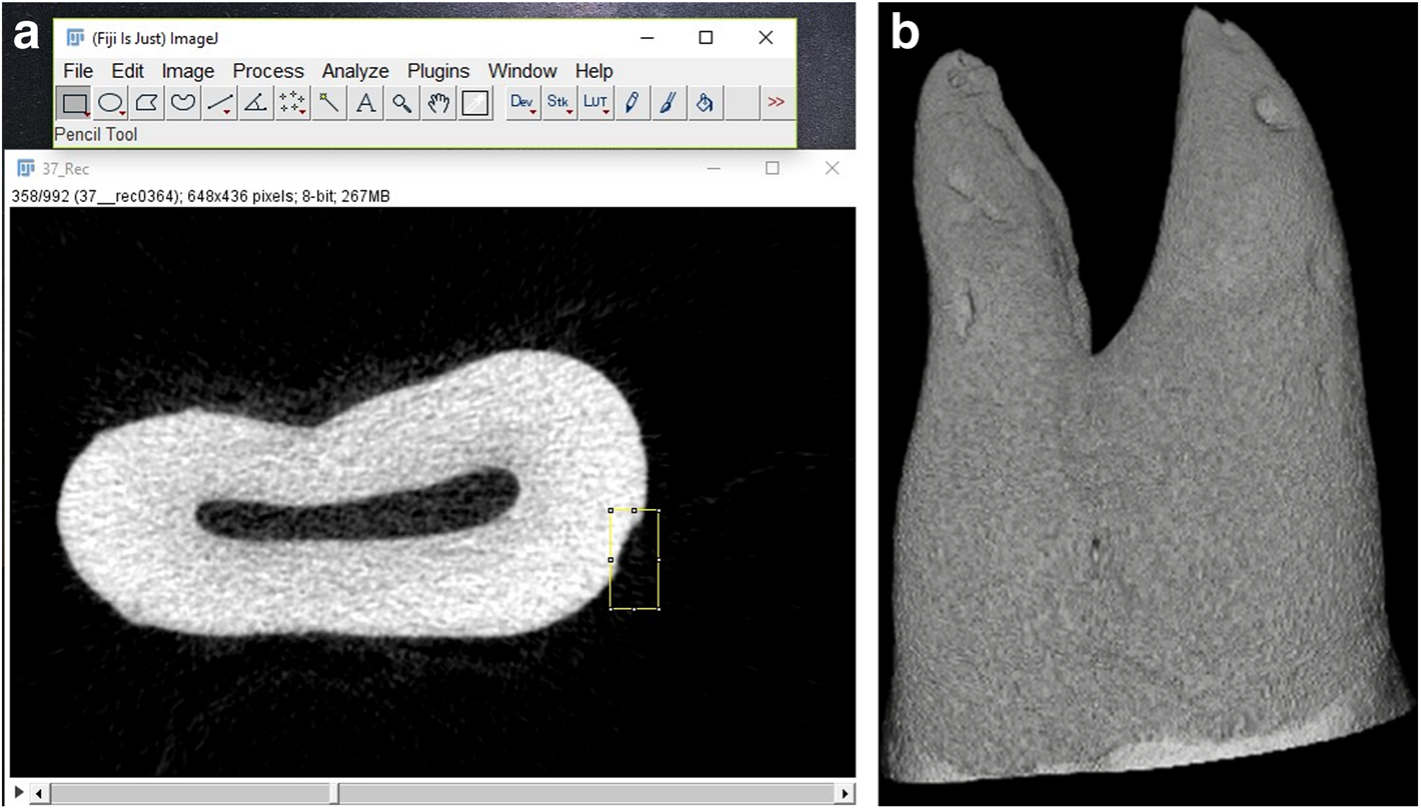
Volume measurement with ImageJ and micro-CT image

Scanning of the root surfaces was applied using the SkyScan-1172 X-ray micro-computed tomography (CT) device (SkyScan, Aartselaar, Belgium). To calculate the volume of the resorption craters isolated on the axial slices, the open-source Fiji (ImageJ) software was used. Using the convex hull module in the software, a line was drawn joining the edges of the resorption craters, and the area below the line was calculated as the volume (Fig. 4). Volumetric changes in the craters on the root surfaces were evaluated both locally and totally.

The data were statistically analyzed using SPSS version 24.0 (IBM Corp., Armonk, NY, USA). The Shapiro-Wilk test was used to test for normal distribution. The Wilcoxon test was applied to the within-group comparisons of the resorption volumes formed at different levels (cervical, mid, apical) on different surfaces of the root (buccal, palatal, mesial, and distal). The Mann–Whitney U test was used for comparison between the groups.

## Results

Intra-group comparisons of the vibration and control sides for the no-force and force groups are shown in Tables 1 and 2. The mean total root resorption amount was not found statistically significant between the vibration and control sides (*P* > 0.05). The comparison of different surfaces (buccal, palatal, mesial, and distal) and levels (cervical, mid, and apical) for the no-force and force groups did not show any significant difference between the vibration and control sides (*P* > 0.05).

**Table 1:** Intra-group comparisons of the vibration and control sides within the no-force group

Variables	Vibration (N = 10)	Control (N = 10)	Z	*P*
	Mean ± SD/mm^3^	Mean ± SD/mm^3^		
Buccal	0.000	0.019 ± 0.054	− 1.604	0.109
Palatal	0.002 ± 0.005	0.009 ± 0.03	− 1.000	0.317
Mesial	0.015 ± 0.047	0.000	− 1.000	0.317
Distal	0.005 ± 0.015	0.002 ± 0.003	− 1.214	0.225
Cervical	0.000	0.028 ± 0.084	− 1.826	0.068
Mid	0.018 ± 0.056	0.001 ± 0.002	− 1.342	0.180
Apical	0.003 ± 0.009	0.002 ± 0.006	− 0.447	0.655
Total	0.017 ± 0.337	0.031 ± 0.085	− 0.314	0.753

Wilcoxon signed-rank test, *p ≤ 0.05 statistical significance at level α = 0.05. *SD* standard deviation

**Table 2:** Intra-group comparisons of the vibration and control sides within the force group

Variables	Vibration (N = 10)	Control (N = 10)	Z	*P*
	Mean ± SD/mm^3^	Mean ± SD/mm^3^		
Buccal	0.161 ± 0.107	0.176 ± 0.192	− 0.561	0.575
Palatal	0.074 ± 0.078	0.062 ± 0.056	− 0.255	0.799
Mesial	0.144 ± 0.089	0.142 ± 0.175	− 0.051	0.959
Distal	0.098 ± 0.085	0.082 ± 0.078	− 0.663	0.508
Cervical	0.201 ± 0.112	0.186 ± 0.148	− 0.255	0.799
Mid	0.186 ± 0.154	0.21 ± 0.231	− 0.255	0.799
Apical	0.088 ± 0.085	0.066 ± 0.06	− 0.764	0.445
Total	0.476 ± 0.236	0.462 ± 0.337	− 0.051	0.959

Wilcoxon signed-rank test, *p ≤ 0.05 statistical significance at level α = 0.05. *SD* standard deviation

The comparison of root resorption of the vibration side for the no-force and force group are presented in Table 3. The mean total root resorption for the vibration side was 0.025 mm^3^ in the no-force group and 0.475 mm^3^ in the force group. A statistically significant difference was found (*P* ≤ 0.001) between the no-force and force groups. A statistically significant root resorption was found for all surfaces (buccal, palatal, mesial, and distal) and levels (cervical, mid, and apical) of the force group when compared to the no-force group (*P* ≤ 0.001).

**Table 3:** Comparisons of root resorption between the groups on the vibration side

Variables	No-force (N = 10)	Force (N = 10)	U	*P*
	Mean ± SD/mm^3^	Mean ± SD/mm^3^		
Buccal	0.000	0.161 ± 0.107	0.000	< 0.001*
Palatal	0.002 ± 0.005	0.074 ± 0.078	11.000	0.001*
Mesial	0.015 ± 0.047	0.144 ± 0.089	8.500	0.001*
Distal	0.005 ± 0.015	0.098 ± 0.085	6.000	0.001*
Cervical	0.000	0.201 ± 0.112	5.000	< 0.001*
Mid	0.018 ± 0.056	0.186 ± 0.154	7.000	0.001*
Apical	0.003 ± 0.009	0.088 ± 0.085	7.500	0.001*
Total	0.017 ± 0.337	0.476 ± 0.236	0.000	< 0.001*

Mann–Whitney U test, *p ≤ 0.05 statistical significance at level α = 0.05. *SD* standard deviation

The statistical data of the comparisons between the force and no-force groups in respect to root resorption of the control side, i.e., mechanical vibration was not applied, are presented in Table 4. The mean total root resorption was measured as 0.462 mm^3^ for the force group and 0.031 mm^3^ for the no-force group. The difference between the groups was statistically significant (*P* ≤ 0.001). Similar to the vibration side, more root resorption was obtained for the control side in the force group when compared to the no-force group (*P* < 0.05).

**Table 4:** Comparisons of root resorption between the groups on the control side

Variables	No-force (N = 10)	Force (N = 10)	U	*P*
	Mean ± SD/mm^3^	Mean ± SD/mm^3^		
Buccal	0.019 ± 0.054	0.176 ± 0.192	8.000	0.001*
Palatal	0.009 ± 0.03	0.062 ± 0.056	12.500	0.002*
Mesial	0.000	0.142 ± 0.175	10.000	0.001*
Distal	0.002 ± 0.003	0.082 ± 0.078	13.000	0.003*
Cervical	0.028 ± 0.084	0.186 ± 0.148	7.000	0.001*
Mid	0.001 ± 0.002	0.21 ± 0.231	0.000	< 0.001*
Apical	0.002 ± 0.006	0.066 ± 0.06	6.500	< 0.001*
Total	0.031 ± 0.085	0.462 ± 0.337	2.000	< 0.001*

Wilcoxon signed-rank test, *p ≤ 0.05 statistical significance at level α = 0.05. *SD* standard deviation

## Discussion

The comparison of the mean total resorption for the force and no-force group did not show a statistically significant difference between the vibration and control sides (*P* > 0.05). Therefore, the null hypothesis was accepted. The alternative hypothesis was also accepted, because a significant difference was found between the force and no-force group (*P* < .001).

In the present study, occlusal and orthodontic forces were removed, and only the effect of vibration on root resorption was investigated, because the idea that vibration during orthodontic force application should prevent PDL compression with increasing bone remodeling and turnover is reasonable. Additionally, with the medical use of vibration, bone remodeling and turnover are increased, thereby accelerating cellular differentiation [10, 11]. Dental use of mechanical vibration may provide the same effect on cementum which is similar to the mineralized structure of the bone. Instead, mechanical vibration could cause extra root resorption due to the vibration frequency or the physiological and cellular differences between the bone and the cementum. However, in the present study, there was no statistically significant difference for resorption between the control and vibration sides of the no-force group (*P* = 0.753).

Nishimura et al. [13] evaluated the effects of mechanical vibration by examining the cellular and molecular mechanisms of the PDL responses. In the control group of 6-week-old male Wistar rats, force was applied to the buccal side of the first molar teeth for 21 days using a spring, and the amount of tooth movement was measured. The results of the study showed that the application of vibration accelerated orthodontic tooth movement by increasing the RANKL level within the PDL, but there was no effect on root resorption. In a study by DiBiase et al. [21], the effect of vibration application with the Acceledent device on OIIRR was examined using periapical radiographs. Mechanical vibration during the alignment phase of fixed orthodontic treatment did not affect OIIRR associated with the maxillary central incisors.

In the current study, the comparison of total root resorption volume of the vibration side for the different surfaces and for the different levels of the root with the control side, i.e., force application side, did not present any statistically significant difference. These results were consistent with the findings of the studies by Nishimura et al. [13] and DiBiase et al. [21]. This can be interpreted as vibration in the presence of force that does not affect the amount of resorption.

Paetyangkul et al. [25] examined the amount of root resorption after a 12-week application of controlled force on the buccal direction. Examinations were made with micro-CT of 40 maxillary and mandibular first premolar teeth extracted from 10 patients after the application of light force (25 g) on one side and heavy force (225 g) on the other side. There was significantly less root resorption in the light force application when compared to the heavy force application. In another study, similar force application mechanics and micro-CT were used and reported that the control groups, i.e., no force application, found fewer and smaller root resorption craters when compared to the other force application groups [2]. Other studies in the literature have shown that the amount of resorption increases with an increase in force [1, 26]. In this study, greater OIIRR was observed in the 150 g force group when compared to the no-force group, with or without vibration (*P* < 0.05).

Nishimura et al. [13] applied 60-Hz mechanical vibration to rat first molars while Liao et al. [20] applied 50-Hz vibration to the buccal surfaces of the canine teeth. Furthermore, in numerous studies [15–17, 19, 21], the application procedure and the frequency value of the AcceleDent device (application of 30-Hz mechanical vibration for 20 min/day) is different from the aforementioned studies. A systematic review stated that vibration values reported in the literature related to mechanical vibration varied to a significant degree, and it was reported to be difficult to access definitive results related to the most effective vibration values [9]. However, Nishimura et al. [13] measured the natural mechanical vibration frequency of the rat as 60 Hz. Therefore, the vibration protocol in the current study was 50 Hz for 10 min/days.

As in similar previous studies [20, 23], a force of 150 g was applied with a 0.017 × 0.025 in. TMA cantilever spring in the current study. After the application of this active 150-g force, resorption craters start to be seen within 10–35 days [27]. Human and animal research showed that the degree of root resorption increases as the experimental period progresses [28, 29]. Paetyangkul et al. did not find any significant difference between buccal force application periods of 4 and 8 weeks; however, the amount of root resorption significantly increased from 8 to 12 weeks [30]. Nevertheless, when the literature is reviewed, the effect of mechanical vibration on root resorption of human teeth has not been fully understood [9]. Thus, a 12-week period was designated to clearly reveal the effects of mechanical vibration on resorption.

If surface resorption is not severe, OIIRR can be detected only as root shortening on panoramic or periapical radiographs [1]. Scanning electron microscope (SEM) has been used in several studies to investigate OIIRR [27, 29]. Although it provides detailed information about the mineralized structure of cement and the resorption crater, the difficulty of creating a sample and the greater number of steps to form an image with this method make the evaluation of a large number of samples difficult and time-consuming [31]. Micro-CT is a variation of a medical CT scanning system that visualizes the internal micro-structure of a material at a non-destructive high spatial resolution [32]. Three-dimensional images obtained from 2-dimensional slices facilitate the identification of resorption craters [31] and can be used to quantify and qualify root surface resorption craters [2]. The dose and length of exposure of radiation are the limitations of this technology for patient applications. However, these limitations are not valid for non-living objects, such as extracted teeth, where a higher radiation dose and longer scanning time can be used.

In this study, OIIRR in the buccal-cervical and palatal-apical regions matched with the expected pressure areas within the PDL. The resorption on the buccal-cervical region was consistent with a buccal tipping movement as shown in previous studies [25, 33], but the palatal-apical region was not consistent. This may be related with our appliance design, which does not generate occlusal forces that cause resorption with apical stress accumulation. The resorption on the mesial and distal surfaces is not consistent with a buccal tipping movement, implying a rotational or torqueing component of the tooth movement [9]. This is quite likely considering the design of the force delivery system which was a compromise between simulating the actual clinical setting and decreasing its complexity for patient comfort. This force delivery system may have affected the locations and amounts of the root resorption craters. Future research should aim to isolate individual tooth movements. Another limitation of this study is that factors such as buccal bone thickness and the tendency to OIIRR depending on individual factors cannot be eliminated. However, similar clinical studies also have these factors due to the heredity of humans.

## Conclusion

This study showed that mechanical vibration has no effect on the amount of root resorption with or without force application. Nevertheless, there was a significant difference in root resorption between the groups in which orthodontic force (150 g) was applied and the group in which no force was applied.

## Data Availability

The data supporting the study can be obtained directly from the corresponding author.

## Abbreviations

OIIRR: Orthodontically induced inflammatory root resorption
TMA: Titanium-molybdenum alloy
RANKL: Receptor activator of nuclear factor kappa-Β ligand
PDL: Periodontal ligament
CT: Computed tomography
SEM: Scanning electron microscope

## References

[1] Chan E, Darendeliler M (2004). Exploring the third dimension in root resorption. Orthod Craniofacial Res.

[2] Harris DA, Jones AS, Darendeliler MA (2006). Physical properties of root cementum: part 8. Volumetric analysis of root resorption craters after application of controlled intrusive light and heavy orthodontic forces: a microcomputed tomography scan study. Am J Orthod Dentofac Orthop.

[3] Brezniak N, Wasserstein A (1993). Root resorption after orthodontic treatment: part 1. Literature review. Am J Orthod Dentofac Orthop.

[4] Lund H, Gröndahl K, Hansen K, Gröndahl H-G (2011). Apical root resorption during orthodontic treatment: a prospective study using cone beam CT. Angle Orthod.

[5] Remington DN, Joondeph DR, Årtun J, Riedel RA, Chapko MK (1989). Long-term evaluation of root resorption occurring during orthodontic treatment. Am J Orthod Dentofac Orthop.

[6] Blake M, Woodside D, Pharoah M (1995). A radiographic comparison of apical root resorption after orthodontic treatment with the edgewise and Speed appliances. Am J Orthod Dentofac Orthop.

[7] Killiany DM (2002). Root resorption caused by orthodontic treatment: review of literature from 1998 to 2001 for evidence. Prog Orthod.

[8] Lyu C, Zhang L, Zou S (2019). The effectiveness of supplemental vibrational force on enhancing orthodontic treatment. A systematic review. Eur J Orthod.

[9] Prisby RD, Lafage-Proust M-H, Malaval L, Belli A, Vico L (2008). Effects of whole body vibration on the skeleton and other organ systems in man and animal models: what we know and what we need to know. Ageing Res Rev.

[10] Sakamoto M, Fukunaga T, Sasaki K, Seiryu M, Yoshizawa M, Takeshita N, Takano-Yamamoto T (2019). Vibration enhances osteoclastogenesis by inducing RANKL expression via NF-κB signaling in osteocytes. Bone..

[11] Leung KS, Shi HF, Cheung WH (2009). Low-magnitude high-frequency vibration accelerates callus formation, mineralization, and fracture healing in rats. J Orthop Res.

[12] Darendeliler MA, Zea A, Shen G, Zoellner H (2007). Effects of pulsed electromagnetic field vibration on tooth movement induced by magnetic and mechanical forces: a preliminary study. Aust Dent J.

[13] Nishimura M, Chiba M, Ohashi T (2008). Periodontal tissue activation by vibration: intermittent stimulation by resonance vibration accelerates experimental tooth movement in rats. Am J Orthod Dentofac Orthop.

[14] Celebi F, Turk T, Bicakci AA (2019). Effects of low-level laser therapy and mechanical vibration on orthodontic pain caused by initial archwire. Am J Orthod Dentofac Orthop.

[15] Miles P, Fisher E (2016). Assessment of the changes in arch perimeter and irregularity in the mandibular arch during initial alignment with the AcceleDent Aura appliance vs no appliance in adolescents: a single-blind randomized clinical trial. Am J Orthod Dentofac Orthop.

[16] Miles P, Fisher E, Pandis N (2018). Assessment of the rate of premolar extraction space closure in the maxillary arch with the AcceleDent Aura appliance vs no appliance in adolescents: a single-blind randomized clinical trial. Am J Orthod Dentofac Orthop.

[17] DiBiase AT, Woodhouse NR, Papageorgiou SN (2018). Effects of supplemental vibrational force on space closure, treatment duration, and occlusal outcome: a multicenter randomized clinical trial. Am J Orthod Dentofac Orthop.

[18] Miles P, Smith H, Weyant R, Rinchuse DJ (2012). The effects of a vibrational appliance on tooth movement and patient discomfort: a prospective randomised clinical trial. Aust Orthod J.

[19] Woodhouse NR, DiBiase AT, Papageorgiou SN, et al. Supplemental vibrational force does not reduce pain experience during initial alignment with fixed orthodontic appliances: a multicenter randomized clinical trial. Sci Rep. 2015;5(1). 10.1038/srep17224. 10.1038/srep17224 PMC4661602 26610843

[20] Liao Z, Elekdag-Turk S, Turk T, Grove J, Dalci O, Chen J, Zheng K, Ali Darendeliler M, Swain M, Li Q (2017). Computational and clinical investigation on the role of mechanical vibration on orthodontic tooth movement. J Biomech.

[21] DiBiase AT, Woodhouse NR, Papageorgiou SN (2016). Effect of supplemental vibrational force on orthodontically induced inflammatory root resorption: a multicenter randomized clinical trial. Am J Orthod Dentofac Orthop.

[22] Leach H, Ireland A, Whaites E (2001). Radiographic diagnosis of root resorption in relation to orthodontics. Br Dent J.

[23] Aras B, Cheng LL, Turk T, Elekdag-Turk S, Jones AS, Darendeliler MA (2012). Physical properties of root cementum: part 23. Effects of 2 or 3 weekly reactivated continuous or intermittent orthodontic forces on root resorption and tooth movement: a microcomputed tomography study. Am J Orthod Dentofac Orthop.

[24] Khaw CMA, Dalci O, Foley M, Petocz P, Darendeliler MA, Papadopoulou AK (2018). Physical properties of root cementum: part 27. Effect of low-level laser therapy on the repair of orthodontically induced inflammatory root resorption: a double-blind, split-mouth, randomized controlled clinical trial. Am J Orthod Dentofac Orthop.

[25] Paetyangkul A, Türk T, Elekdağ-Türk S, Jones AS, Petocz P, Darendeliler MA (2009). Physical properties of root cementum: part 14. The amount of root resorption after force application for 12 weeks on maxillary and mandibular premolars: a microcomputed-tomography study. Am J Orthod Dentofac Orthop.

[26] Chan E, Darendeliler MA (2006). Physical properties of root cementum: part 7. Extent of root resorption under areas of compression and tension. Am J Orthod Dentofac Orthop.

[27] Harry M, Sims M (1982). Root resorption in bicuspid intrusion: a scanning electron microscope study. Angle Orthod.

[28] Acar A, Canyürek Ü, Kocaaga M, Erverdi N (1999). Continuous vs. discontinuous force application and root resorption. Angle Orthod.

[29] Kurol J, Owman-Moll P, Lundgren D (1996). Time-related root resorption after application of a controlled continuous orthodontic force. Am J Orthod Dentofac Orthop.

[30] Paetyangkul A, Türk T, Elekdağ-Türk S, Jones AS, Petocz P, Cheng LL (2011). Physical properties of root cementum: part 16. Comparisons of root resorption and resorption craters after the application of light and heavy continuous and controlled orthodontic forces for 4, 8, and 12 weeks. Am J Orthod Dentofac Orthop.

[31] Hohmann A, Wolfram U, Geiger M (2007). Periodontal ligament hydrostatic pressure with areas of root resorption after application of a continuous torque moment: a study using identical extracted maxillary human premolars. Angle Orthod.

[32] Stock S (1999). X-ray microtomography of materials. Int Mater Rev.

[33] Cheng LL, Türk T, Elekdağ-Türk S, Jones AS, Petocz P, Darendeliler MA (2009). Physical properties of root cementum: part 13. Repair of root resorption 4 and 8 weeks after the application of continuous light and heavy forces for 4 weeks: a microcomputed-tomography study. Am J Orthod Dentofac Orthop.

